# DNA Barcoding of Malagasy Rosewoods: Towards a Molecular Identification of CITES-Listed *Dalbergia* Species

**DOI:** 10.1371/journal.pone.0157881

**Published:** 2016-06-30

**Authors:** Sonja Hassold, Porter P. Lowry, Martin R. Bauert, Annick Razafintsalama, Lolona Ramamonjisoa, Alex Widmer

**Affiliations:** 1 Institute of Integrative Biology, ETH Zurich, Zurich, Switzerland; 2 Missouri Botanical Garden, St. Louis, Missouri, United States of America; 3 ISYEB (UMR 7205), Département Systématique et Evolution, Muséum National d’Histoire Naturelle, Paris, France; 4 Zoo Zurich AG, Zurich, Switzerland; 5 Silo National des Graines Forestiers, Ambatobe, Antananarivo, Madagascar; Biodiversity Research Center, Academia Sinica, TAIWAN

## Abstract

Illegal selective logging of tropical timber is of increasing concern worldwide. Madagascar is a biodiversity hotspot and home to some of the world’s most sought after tropical timber species. Malagasy rosewoods belong to the genus *Dalbergia* (Fabaceae), which is highly diverse and has a pantropical distribution, but these timber species are among the most threatened as a consequence of intensive illegal selective logging and deforestation. Reliable identification of *Dalbergia* species from Madagascar is important for law enforcement but is almost impossible without fertile plant material, which is often unavailable during forest inventories or when attempting to identify logged trees of cut wood. DNA barcoding has been promoted as a promising tool for species identification in such cases. In this study we tested whether DNA barcoding with partial sequences of three plastid markers (*mat*K, *rbc*L and *trn*L (UAA)) can distinguish between *Dalbergia* from Madagascar and from other areas of its distributional range, and whether Malagasy species can be distinguished from one another. Phylogenetic analyses revealed that the Malagasy *Dalbergia* species studied form two monophyletic groups, each containing two subgroups, only one of which corresponds to a single species. We characterized diagnostic polymorphisms in the three DNA barcoding markers that allow rapid discrimination between *Dalbergia* from Madagascar and from other areas of its distribution range. Species identification success based on individual barcoding markers or combinations was poor, whereas subgroup identification success was much higher (up to 98%), revealing both the value and limitations of a DNA barcoding approach for the identification of closely related Malagasy rosewoods.

## Introduction

In recent years human-caused pressure on wildlife has increased dramatically, most notably in the tropics, as a consequence of increasing demand for animal and plant products and the resulting high prices they command on the international market. A wide diversity of species are affected, but only a few are widely known, such as elephants and rhinos, which are heavily poached for their valuable ivory tusks and horns, respectively [1,2]. Plant species are also severely impacted by international trade, including many tropical hardwood trees such as mahogany and ebony, which are in high demand for their valuable timber [3,4].

Madagascar is renowned as one of the world’s most important tropical biodiversity hotspots [5] but its remarkable fauna and flora are under growing pressure as a consequence of slash and burn agriculture, charcoal production, and selective illegal logging [6–10]. This situation was aggravated by the political coup that took place in Madagascar in 2009 and the following suspension of most international financial assistance to the Malagasy government [11]. The ensuing increased political instability was accompanied by growing governmental corruption and a worsening of Madagascar’s already widespread poverty [12,13]. This created a favorable environment for the massive increase in illegal logging that has plagued the country over the last several years [14,15]. Randriamalala & Liu [13] estimated that almost all illegally logged timber in Madagascar is exported to China, primarily through ports in eastern Africa. The genus *Dalbergia* provides a prime example of Malagasy timber species that are being heavily impacted by excessive illegal logging to feed a highly profitable, demand-driven hardwood market [14,16].

*Dalbergia*, a pantropical member of the species-rich family Fabaceae with representatives also occurring in Central and South America, continental Africa, and Asia, has approximately 140 species worldwide, 47 of which are endemic to Madagascar, where they grow in a wide range of habitats, from humid evergreen rainforest to seasonal deciduous and dry forests [17]. Several of these endemic species are among the world’s most valuable timbers, with sales reaching hundreds of millions of dollars on the international market [16,18]. The heartwood of exploited Malagasy *Dalbergia* species is highly distinctive: it is exceptionally dense, non-porous, and durable, exhibits remarkable variation in color, and is highly suitable for carving and for intricate woodwork, all of which make it in high demand, especially for the production of furniture and musical instruments [13,16]. Wood obtained from members of the genus in Madagascar is placed in two categories, rosewood and *palissandre*, which can be recognized based on coloration, ranging from dark red and black patterned hardwood to brown or purple scales.

While exploited species can easily be assigned to rosewood and *palissandre* based on wood quality and coloration, species identification is more difficult. The taxonomy of Malagasy *Dalbergia* has been the subject of two important studies [17,19], and species can be distinguished from one another by characters of their flowers and fruits. These studies, however, contain limited information on other important features of species such as details of the shape of leaves and leaflets, which could aid species identification, or information on age at reproduction or mating system, which are relevant to assess species vulnerability to illegal logging. As a consequence, while the *Dalbergia* species occurring in Madagascar can be distinguished from one another by characters of their flowers and fruits [17,19], they are practically impossible to tell apart without reproductive structures (i.e., when only leaves are available) or based on their wood alone. Also, while one can readily distinguish between rosewood and *palissandre*, it is not clear to which botanical species they belong. This is problematic for both the management and conservation of *Dalbergia* species in Madagascar because species represent the fundamental unit in conservation [16,17]. Moreover, because species growing in other parts of the world also produce wood with red colored heartwood that is so characteristic of rosewood [20,21], identification and determination of the origin of material is exceedingly difficult.

In an effort to reduce and ultimately halt illegal logging and trade in Malagasy rosewoods, a proposal from the Malagasy government led to the listing in 2013 of all Malagasy species of *Dalbergia* on CITES Appendix II [22]. In addition, a ban on exportation of rosewood established by the Malagasy government currently prohibits any international trade of these species (decree no. 2010–141). Despite these efforts, however, illegal trade of *Dalbergia* from Madagascar has continued unabated as permits are frequently falsified to indicate that the wood being shipped has a different origin (such as continental Africa) or to identify the wood using a broad name that cannot be associated with any particular genus or species in order to avoid difficulties at checkpoints in transfer ports. The effective enforcement of CITES and of local laws and regulations requires that the identity of wood being traded can be accurately validated, that the declared origin can be verified, and that this can be done quickly and efficiently using expert knowledge and appropriate, reliable techniques. To date timber identification by customs officials is mainly done using wood anatomical characters and in rare instances stable isotopes. Both techniques are, however, inadequate to identify individual timber species, although they may in some cases be sufficient to assess the genus and geographical origin of the wood [23].

Molecular identification techniques such as DNA barcoding could serve as another tool to help identify and trace rosewoods and other valuable hardwoods. Over the last several years a number of studies have demonstrated the power of DNA barcoding for species identification and forensic analyses of protected plant species [e.g., Aubriot *et al*. [24], Finkeldey *et al*. [25], Lahaye *et al*. [26], Lowe & Cross [23] and Muellner *et al*. [27]]. Chloroplast (cp) DNA markers, including *mat*K, *rbc*L and *trn*L (UAA), have been proposed as standard DNA barcoding markers [28] and have been used successfully in some plant groups to address questions of species identification, phylogenetic relationships and to infer sample origin [29,30,31,32]. Recent studies using Asian and Indian *Dalbergia* species showed the potential of these markers for species identification [33–35]. In this study, we explore the potential use of cp DNA markers for barcoding of *Dalbergia* species from eastern Madagascar. More specifically, we address the following questions: 1) can we use standard DNA barcoding markers to distinguish Malagasy from non-Malagasy *Dalbergia* species?; 2) do *Dalbergia* species from Madagascar differ from one another in their DNA barcoding sequences? and; 3) with what accuracy can we identify *Dalbergia* species from Madagascar using DNA barcoding?

## Materials and Methods

### Species sampling

**Fig 1:**
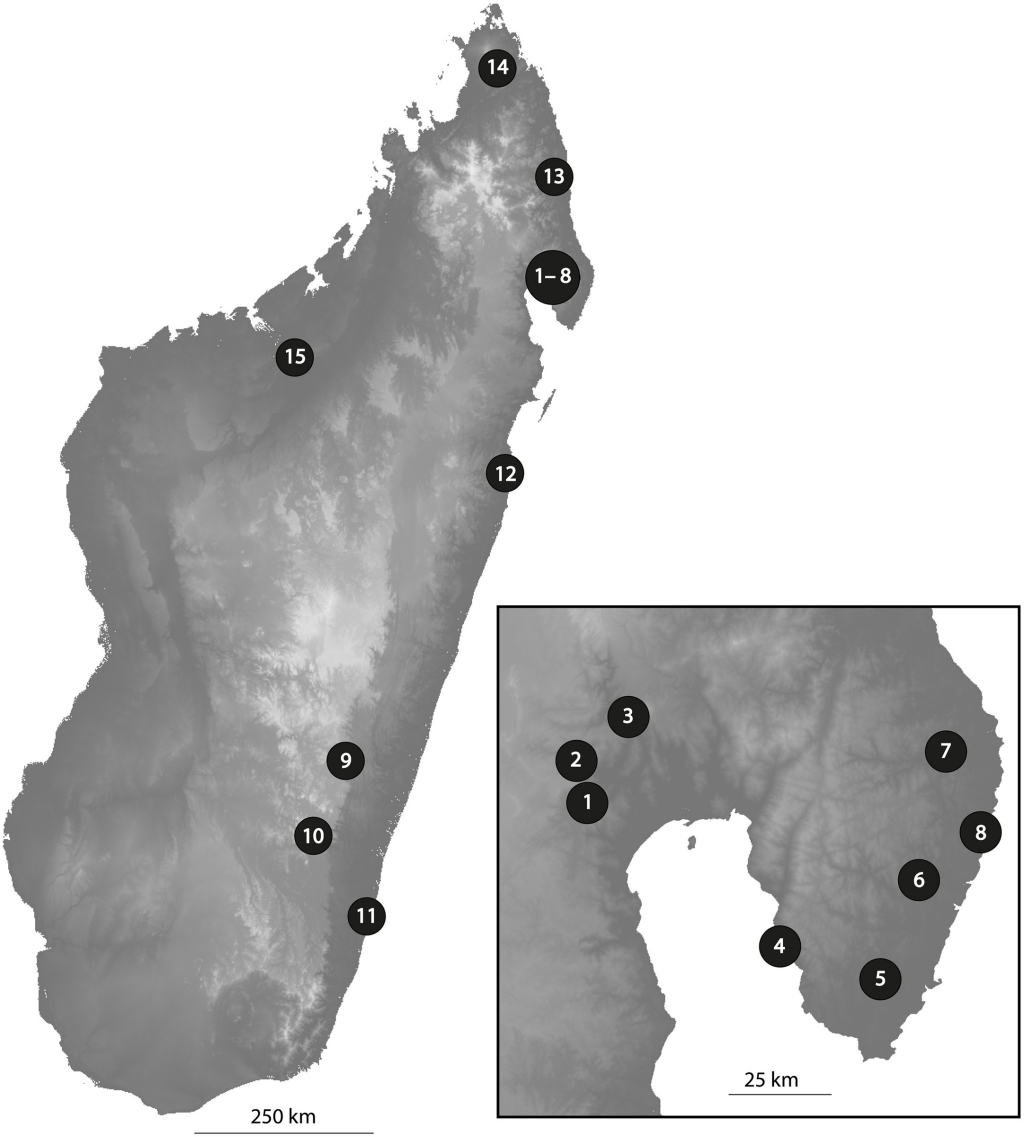
Map of Madagascar illustrating the geographic positions of sampling sites. The inset in the bottom right corner shows the Bay of Antongil. Gray scale values denote elevation, with lighter color indicating higher elevations. More detailed information about sampling locations is given in Table 1.

We collected 121 fully vouchered *Dalbergia* leaf samples from 15 study areas in Madagascar between 2010 and 2014 (Fig 1, Table 1 and S1 Table). Our collection efforts focused on Northeastern Madagascar and especially the Masoala peninsula, where *Dalbergia* species diversity is high [16,17] and where the most extensive illegal logging activities occurred in protected areas in 2011 and preceding years [11]. We subsequently expanded the study area southwards to provide better coverage of species distribution ranges and to assess intraspecific variation in barcoding markers.

**Table 1:** Geographic positions of sampling sites in Madagascar. Site numbers are as in Fig 1. The coordinates are averaged over all samples collected at a given site.

Number	Province	Location	Latitude	Longitude
1	Tamatave	Anjiahely	-15.3961	49.5247
2	Tamatave	Mangabe, Ambinanitelo	-15.3063	49.5004
3	Tamatave	Andaparaty	-15.2028	49.6198
4	Tamatave	Tampolo	-15.7277	49.9665
5	Antsiranana	Magniria, Vinanivao	-15.8036	50.1979
6	Antsiranana	Sahabe, Ampanavoana	-15.5734	50.2850
7	Antsiranana	Anjia/Sahafary, Ambohitralanana	-15.2836	50.3505
8	Antsiranana	Ratsinarana	-15.4650	50.4280
9	Finarantsoa	Ranomafana	-21.2636	47.4292
10	Finarantsoa	Andringitra	-22.1885	47.0318
11	Finarantsoa	Farafangana	-23.1667	47.6833
12	Tamatave	Betampona Reserve, Foulpointe	-17.7500	49.3778
13	Antsiranana	Makirovana, Sambava	-14.1000	49.9833
14	Antsiranana	Ankarana Reserve/Sahafary	-12.7869	49.2885
15	Mahajanga	Ankarafantsika Reserve	-16.3183	46.8106

Leaf samples were stored in silica gel and herbarium vouchers were pressed in the field and stored in alcohol (70%) to prevent decomposition prior to being dried. In addition we included samples from four taxa occurring mainly in western Madagascar that were collected in April 2014. Madagascar’s Ministry of Environment, Ecology, Sea and Forests issued permits for sampling at all locations under permits N°180/11/MEF/SG/DGF/DCB.SAP/SCBSE and N°222/13/MEF/SG/DGF/DCB.SAP/SCBSE. The same authority also issued export permits for all collected samples (034C_EV01/MG12, 307C_EV06/MG12, and 201N_EV11/MG13).

For reliable identification of *Dalbergia* species in the field it is preferable to collect material with flowers and fruits, as leaf morphology alone is rarely if ever adequate when using available identification tools. Sterile vouchers were identified based on results from a detailed leaf-morphometric analysis (Hassold *et al*. unpublished results) and comparisons with type specimens and herbarium vouchers identified on the basis of flowers and fruits at the Herbarium Musei Parisiensis (P) in Paris, France. Our 121 fully vouchered Malagasy samples (S1 Table), of which only four were fertile, represent 15 *Dalbergia* taxa (including infraspecific entities, but hereafter referred to as species) from Madagascar. To date, 16 *Dalbergia* species have been reported from Eastern Madagascar of which we were able to collect 10 (63%). The six species that we did not encounter during our fieldwork are extremely rare and only known from a small number of locations [17, 19]. Details on sample origin and identity are provided in Table 1 and S1 Table. To test whether the sampled *Dalbergia* species from Madagascar form a monophyletic group compared to non-Malagasy species and to evaluate whether DNA barcoding can be used to discriminate between Malagasy *Dalbergia* and non-Malagasy members of the genus, we included 41 samples of other *Dalbergia* from across the distribution range of the genus: Bhutan, Bolivia, Brazil, China, India, Mexico, Mozambique, South Africa, Tanzania, Thailand and Vietnam (S1 Table). As outgroup for phylogenetic analyses we used published DNA sequences of *Pterocarpus indicus* Willd. retrieved from GenBank. This species is a member of the *Pterocarpus* clade, which is sister to the *Dalbergia* clade in the newly circumscribed tribe Dalbergieae [30]. The full dataset used in this study contained 162 samples representing 38 *Dalbergia* species plus *Pterocarpus* as outgroup.

### Molecular methods

Genomic DNA was extracted from 15 mg of silica dried leaf material using either a modified CTAB protocol [36] or the DNeasy Plant Mini Kit (QIAGEN, Venlo, The Netherlands). Total DNA was quantified using the *dsDNA BR* assay (life Technologies) for Qubit^™^ 2.0 fluorometer (Invitrogen) and DNA integrity was checked on 1.5% agarose gels.

We first tested multiple primer pairs for 12 chloroplast regions on a subset of the samples (S2 Table). Based on universality, sequencing quality, and discriminatory power inferred from these tests, we selected the two core barcode markers, *mat*K and *rbc*L [28], as well as the *trn*L region (UAA) [37], for further study. The *trn*H–*psb*A region proposed by Hollingsworth *et al*. [29] was not suitable for studying *Dalbergia* species because of poor sequencing quality associated with long mono-nucleotide repeats.

PCR amplifications were carried out in 15 μl total reaction volumes containing 5x PCR buffer (Colorless GoTaq Flexi Buffer; Promega), 25 mM MgCl_2_, 2.5 mM dNTPs, 10 μM of each primer, 0.5 U of Go-Taq Flexi DNA Polymerase (Promega), and 20 ng of template DNA. PCR amplification was performed using the following cycling conditions for all markers: initial denaturation at 95°C (2 min), 32 cycles of 95°C (30 s), annealing at 58°C (30 s) and extension at 72°C (30 s), followed by a final extension at 72°C (5 min). PCR products were EXOSAP purified with 5U exonuclease I (Fermentas) and 0.5 U thermosensitive alkaline phosphatase (Thermo Scientific) at 37°C for 15 min and then at 80°C for another 15 min. We sequenced PCR products in 10 μl reaction volumes with 0.8 μl of BigDye Terminator v3.1 (AB, Life Technologies), 1.6 μl 5x sequencing buffer, 1.6 μl primer (1 μM), 5 μl of ddH_2_O and 1 μl of PCR product. Sequencing reactions were performed using the following conditions: initial denaturation at 94°C (2 min), 45 cycles of 94°C (10 s), annealing at 50°C (5 s) and extension at 60°C (3 min) followed by a final hold at 4°C. We cleaned sequencing products through a Durapore filter plate (Millipore MSHVN4510) loaded with Sephadex^™^ G-50 (GE, Healthcare) and ran the samples at the ETH Zurich Genetic Diversity Centre (GDC) on a 16 capillary sequencer (3130 DNA Analyser, ABI, Life Technologies). All sequences are deposited on the Barcode of Life Data systems (BOLD) (MADA001 to MADA218).

### Data analysis

#### Sequences

Sequence alignment, including trimming, visual inspection and manual adjustments, was performed in Geneious version 7.1.7 [38]. For the trimming parameters we used an error probability of 0.1 per base and a quality threshold of 20. Poor quality base calls at the 5’ and 3’ ends of the sequenced PCR products were removed. Multiple sequence alignment was performed for each gene separately in Geneious using MUSCLE [39] version 3.8.425. Individual alignments were then concatenated to produce a three-gene alignment for all 163 samples (*Dalbergia* and *Pterocarpus*), which can be found on TreeBASE (http://purl.org/phylo/treebase/phylows/study/TB2:S19301). Identical sequences were then pruned from the alignment and only distinct haplotypes were kept for the phylogenetic analysis. The final haplotype data matrix is available at TreeBASE (http://purl.org/phylo/treebase/phylows/study/TB2:S19301). The total length of the concatenated haplotype alignment is 1530 positions (*mat*K: 565 bp, *rbc*L: 510 bp, *trn*L (UAA): 455 bp), including 182 potentially informative positions (*mat*K: 69, *rbc*L: 30, *trn*L (UAA): 83) and 1.4% missing data. Microsatellite repeats were excluded and gaps were treated as missing data for phylogenetic analysis.

#### Phylogenetic analysis

For phylogenetic reconstruction we used Maximum Likelihood (ML) and Bayesian Inference (BI) methods implemented in RAxML [40] version 7.2.8 and MrBayes [41] version 3.2.2, respectively. For the ML analysis, robustness was assessed by running 1,000 fast bootstrap replicates using the GTR + GAMMA nucleotide model. Model selection was based on the Akaike information criterion using the program jModelTest v.2.1.7 [42]. For the BI analysis we partitioned the concatenated alignment and ran a separate GAMMA model for each of the three chloroplast regions. Furthermore, a separate rate multiplier was set for each partition. For the analysis we used 100 million generations, sampled every 100 generations, and we performed four simultaneously independent runs with four cold chains. Twenty-five percent (= 800,000) of the trees were discarded as burn in and posterior probabilities were compiled from the remaining trees. The average standard deviation of split frequencies was below 0.0006. The best-scoring tree was visualized using FigTree v1.4.2 (A. Rambaut; http://tree.bio.ed.ac.uk/software/figtree/) and Dendroscope v3.2.10 [43].

#### Diagnostic polymorphisms

**Fig 2:**
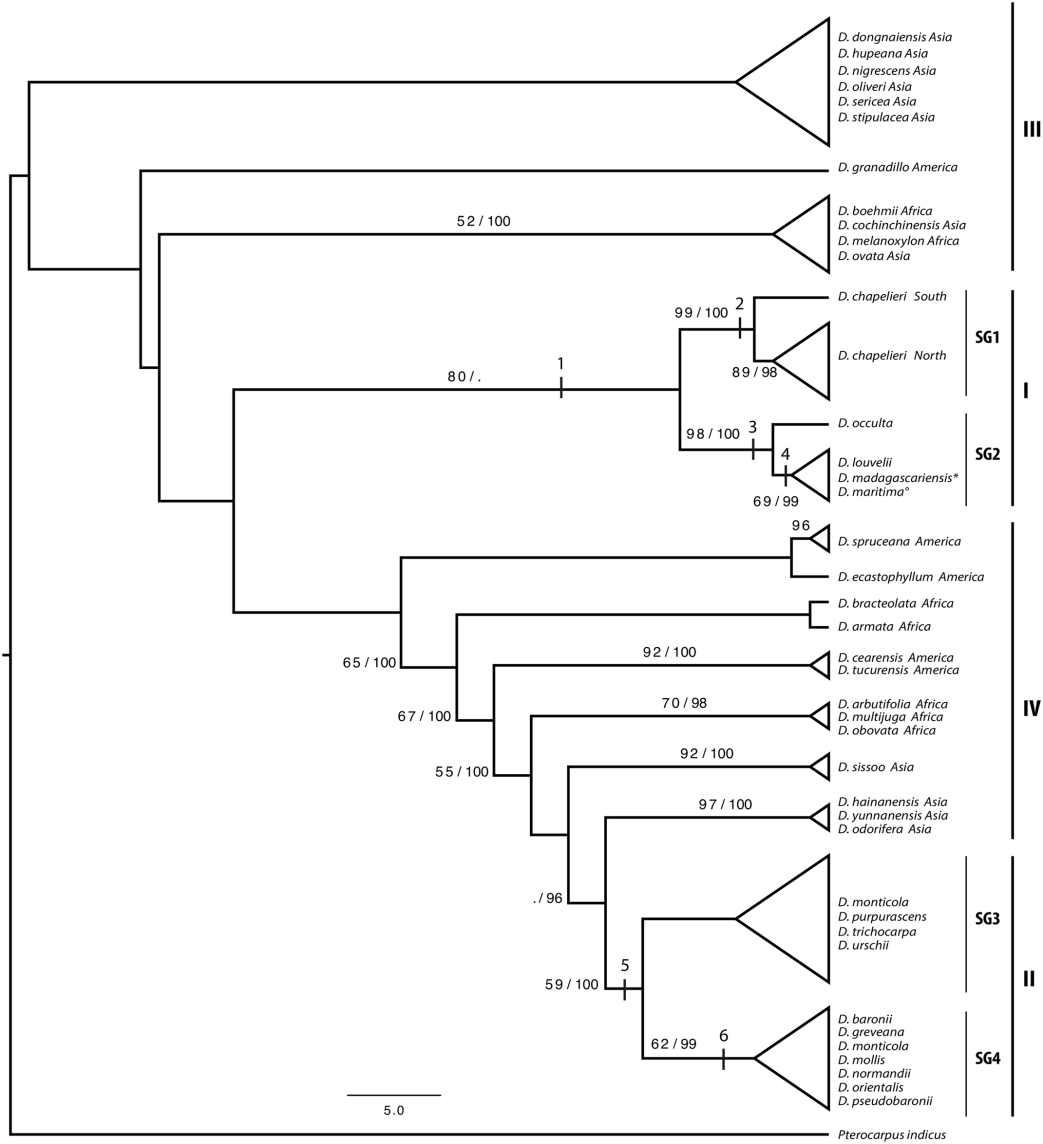
Phylogenetic tree of studied *Dalbergia* species. Malagasy *Dalbergia* species are divided into two main groups, I and II, encompassing three well-supported subgroups (SG1, SG2 and SG4). Only subgroup 1 (SG1) encompasses all samples from a single species, *D*. *chapelieri*, All other subgroups encompass accessions from more than one species. Vertical black bars indicate positions of diagnostic polymorphisms (Table 2) along the phylogenetic tree. Numbers above branches are *bootstrap support values / posterior probabilities* derived from maximum likelihood (ML) and Bayesian analyses, and are shown only when values are above 50 and 94, respectively. Shown is the tree topology derived from the ML analysis. For non-Malagasy *Dalbergia*, the geographic origin (continent) of each species is indicated.* *D*. *madagascariensis* subsp. *antongilensis*; ° *D*. *maritima* var. *pubescens*.

To identify diagnostic polymorphisms that distinguish Malagasy from non-Malagasy *Dalbergia* and that discriminate among taxa from Madagascar we used the CAOS (Characteristic Attribute Organisation System) workbench [44,45]. CAOS not only considers nucleotide substitutions but also takes insertions and deletions into account. We therefore ran the CAOS workbench with our haplotype alignment but did not exclude well aligned indels and gaps. We reduced the total number of 203 diagnostic character states identified by CAOS to the 17 diagnostic character states most relevant to distinguish between Malagasy and non-Malagasy *Dalbergia*, as well as between subgroups of Malagasy taxa (Table 2), following the recommendations of Jörger & Schrödl [46]. The positions of these diagnostic characters along the phylogenetic tree are shown in Fig 2 as black bars.

**Table 2:** Diagnostic polymorphisms for the identification of Malagasy *Dalbergia* based on DNA sequence variation in the plastid genes *mat*K, *rbc*L and *trn*L (UAA). In bold are diagnostic polymorphisms that distinguish Malagasy from non-Malagasy *Dalbergia*.

Groups	Sub-groups	178	205	937	1008	1063	1087	1175	Indel 1	1231	1256	1265	1268	1299	Indel 2	1359	1365	1439
I	SG1	**T**	**G**	A	C	C	**A**	**A**	**1**	G	**G**	T	A	T	1	G	**T**	0
	SG2	C	C	A	**A**	**A**	G	G	**1**	G	C	T	**G**	T	1	G	**T**	**T**
		C	C	A	**A**	**A**	G	G	**1**	G	C	**G**	**G**	T	1	G	**T**	**T**
II	SG3	C	C	A	C	C	G	G	0	**T**	C	T	A	**C**	**0**	**C**	C	0
	SG4	C	C	**G**	C	C	G	G	0	**T**	C	T	A	**C**	**0**	**C**	C	0
III		C	C	A	C	C	G	G	0	G	C	T	A	T	1	G	C	0
IV		C	C	A	C	C	G	G	0	G°	C	T	A	T	1	G	C	0
Out-group		C	C	A	C	A	G	G	0	G	C	T	A	T	1	G	C	0
Bar*		2	2	6	3	3	2	2	1	5	2	4	3	5	5	5	1	3
		*matK*		*rbcL*			*trn*L											

Indel 1: TGAAT (Pos. 1178–1182); Indel 2: TYTHTHDAAT (Pos. 1338–1348);

°position missing in *D*. *arbutifolia*;

* Support for vertical bars in Fig 2.

### Sequence divergence and identification success

We calculated the frequencies of intraspecific and interspecific pairwise p-distances (Kimura 2-parameter distance model) using the program TAXONDNA [47] for each marker (*mat*K, *rbc*L, *trn*L (UAA)) individually and for selected combinations at the species, subgroup and group levels of the full dataset (163 sequences). We used the program SpeciesIdentifier v.1.7.8 of TAXONDNA to evaluate the accuracy of species identification (hereafter referred to as “success rate”) based on barcode sequences from our dataset for each single marker and for selected marker combinations. Because of the occurrence of intraspecific nucleotide variation within several species and of haplotype sharing among some of the species, we also assessed the accuracy of assigning a sample to a certain group or subgroup encompassing multiple species. The proportions of successful identifications were calculated using the ‘Best match’ and ‘Best close match’ criteria according to Meier *et al*. [47].

## Results

### Phylogenetic analysis

Our phylogenetic analyses revealed that the Malagasy *Dalbergia* species included in this study are not monophyletic but rather form two monophyletic groups (groups I and II, Fig 2). None of the endemic *Dalbergia* species from Madagascar group with non-Malagasy members of the genus. Given the limited number of samples representing non-Malagasy *Dalbergia* species, a detailed discussion of their phylogenetic relationships is not warranted and we combined them here into two paraphyletic groups (III and IV, Fig 2). Group III includes six species from Asia that form a clade, *D*. *granadillo* from South America, and a third clade encompassing *D*. *boehmii* and *D*. *melanoxylon* from continental Africa along with two Asian species, *D*. *cochinchinensis* and *D*. *ovata*. Group IV contains four well-supported clades that collectively comprise species from continental Africa, America or Asia. The branches leading to *D*. *armata*, *D*. *bracteolata*, *D*. *ecastophyllum* and *D*. *spruceana* are weakly supported and their phylogenetic positions currently remain unresolved.

Within the two Malagasy groups I and II, our phylogenetic analyses consistently resolved three well-supported monophyletic subgroups (SG1, SG2 and SG4, Fig 2) while SG3 remains poorly supported. Overall, the inferred monophyletic groups poorly correspond to identified species. *Dalbergia chapelieri* is the only Malagasy species for which all collected accessions fell into a single monophyletic subgroup (SG1) encompassing samples from two geographic regions (North versus South). All other subgroups of Malagasy *Dalbergia* (SG2 to SG4) encompass more than one species. In SG2, the single available accession of *D*. *occulta* is sister to a clade encompassing three taxa, *D*. *louvelii*, *D*. *madagascariensis* subsp. *antongilensis* and *D*. *maritima* var. *pubescens*. Subgroup SG3 combines single accessions of *D*. *purpurascens* and *D*. *urschii* that are distinct from samples of *D*. *monticola* (from Ranomafana (site 9, Fig 1) and Andringitra (site 12, Fig 1)) and *D*. *trichocarpa*. Accessions of *D*. *monticola* from Makira (sites 1–3, Fig 1) are placed in SG4 with material of six other species: *D*. *baronii*, *D*. *greveana*, *D*. *mollis*, *D*. *normandii*, *D*. *orientalis* and *D*. *pseudobaronii*. Thus, *Dalbergia monticola*, a species with a wide distribution range, shows genetic differences between geographic regions. Samples collected in the North can be distinguished from samples made in the South.

### Diagnostic polymorphisms

We identified 203 potentially informative polymorphisms for diagnostic purposes distributed across the three barcoding markers used in this study. From these we extracted in total 17 polymorphisms that are diagnostic for Malagasy *Dalbergia* groups I (two polymorphisms) and II (four polymorphisms), as well as subgroups SG1, SG2 and SG4 (five, four and one polymorphisms, respectively). Within SG2 we found one diagnostic polymorphism that allows further differentiation. All diagnostic polymorphisms are indicated in Table 2. The gene region *trn*L (UAA) alone contained 12 informative positions for Malagasy *Dalbergia*. Polymorphisms *Indel1* and 1365 are diagnostic for group I of Malagasy *Dalbergia*. Within this group, nine diagnostic positions from all three markers allow the identification of subgroups SG1 and SG2. Polymorphisms *Indel2*, 1231, 1299 and 1359 are diagnostic for Malagasy *Dalbergia* group II, and within this group, one diagnostic polymorphism (position 937) identifies SG4. Positions of diagnostic polymorphisms are indicated on the phylogenetic tree as numbered vertical bars (Fig 2).

We were not able to use the analysis tool for diagnostic characters from BOLD systems as proposed by Robinson *et al*. [48] and Collins & Cruickshank *et al*. [49] because of the limited species level resolution among the Malagasy samples and the comparatively small number of sequences available for non-Malagasy species.

### Sequence divergence and identification success

Interspecific genetic distances estimated using all three barcoding markers, calculated both individually for each marker and in combination, widely overlapped with intraspecific distances at the species level (Table 3). Genetic distances also widely overlapped among groups and subgroups at levels 1 (Malagasy samples split into groups I and II and non-Malagasy samples divided into monophyletic groups shown in Fig 2) and 2 (Malagasy samples split into SG1-SG4, non-Malagasy samples as in level 1; Table 3). On average 10% of all interspecific distances or genetic distances among groups and subgroups were zero or close to zero (< 0.002).

**Table 3:** Identification success rates and sequence divergence in *Dalbergia* for individual and combined barcoding markers. The samples are analysed at species level and levels 1 and 2 based on the ‘Best match’ and ‘Best close match’ functions of TAXONDNA [47] for individual barcoding markers and selected combinations. Identification success rates for ‘Best match’ and ‘Best close match’ are given as percentages (successfully identified/ambiguous/misidentified samples or /no match). Level 1: Malagasy samples split into groups I and II and non-Malagasy samples divided into monophyletic groups shown in Fig 2. Level 2: Malagasy samples split into SG1 to SG4, non-Malagasy samples as in level 1. Intra- and interspecific distances were calculated using Kimura 2-parameter corrected p-distances between all sequence pairs.

Barcodes	Best match	Best close match	Mean intraspecific distance (range)	Mean interspecific distance (range)
Species level				
*mat*K	21/72/8/-	21/72/6/1	0.0008 (0–0.0126)	0.0088 (0–0.0272)
*rbc*L	10/89/1/-	10/88/1/1	0.0020 (0–0.0099)	0.0073 (0–0.0139)
*trn*L	20/75/5/-	19/69/2/10	0.0010 (0–0.0115)	0.0131 (0–0.0447)
*mat*K + *rbc*L	32/61/7/-	32/60/6/2	0.0014 (0–0.0094)	0.0081 (0–0.0189)
*mat*K + *trn*L	25/65/10/-	24/64/7/5	0.0009 (0–0.0092)	0.0107 (0–0.0306)
*mat*K + *rbc*L + *trn*L	33/56/10/-	32/55/6/7	0.0013 (0–0.0067)	0.0095 (0–0.0235)
Level 1				
*mat*K	95/2/3/-	93/1/2/4	0.0006 (0–0.0144)	0.0094 (0–0.0273)
*rbc*L	60/39/2/-	60/38/2/1	0.0010 (0–0.0099)	0.0078 (0–0.0139)
*trn*L	44/53/3/-	43/53/2/1	0.0005 (0–0.0232)	0.0140 (0–0.0447)
*mat*K + *rbc*L	**98/1/1/-**	**98/1/1/1**	0.0008 (0–0.0094)	0.0086 (0–0.0189)
*mat*K + *trn*L	**98/0/2/-**	**95/0/1/4**	0.0006 (0–0.0171)	0.0114 (0–0.0306)
*mat*K + *rbc*L + *trn*L	**98/0/2/-**	**97/0/1/2**	0.0007 (0–0.0127)	0.0101 (0.0007–0.0235)
Level 2				
*mat*K	30/68/2/-	26/66/1/7	0.0009 (0–0.0144)	0.0113 (0.0018–0.0272)
*rbc*L	45/53/2/-	42/50/2/6	0.0029 (0–0.1186)	0.0085 (0–0.0139)
*trn*L	41/55/4/-	36/53/2/10	0.0022 (0–0.0232)	0.0164 (0–0.0447)
*mat*K + *rbc*L	83/15/1/-	80/14/1/6	0.0019 (0–0.0094)	0.0100 (0.0009–0.0189)
*mat*K + *trn*L	67/29/3/-	60/29/1/9	0.0015 (0–0.0171)	0.0135 (0.0030–0.0306)
*mat*K + *rbc*L + *trn*L	**97/0/3/-**	**91/0/1/7**	0.0020 (0–0.0127)	0.0118 (0.0027–0.0235)

Identification success was lowest (10–33%) at the species level, irrespective of marker combination, and was higher (30–98%) when tested at the level of subgroups and groups, especially when markers were combined (Table 3). The best identification success (95–98%) was obtained when using *mat*K alone and in combination with *rbc*L or *trn*L (UAA) at level 1. Identification success was on average lower (30–96%) when tested at level 2.

## Discussion

This study provides the first chloroplast DNA reference dataset to support the identification of *Dalbergia* species originating from Madagascar. These include traded rosewood species that are of high economic interest and have been severely impacted over recent years by unsustainable, illegal selective logging and the degradation and destruction of their natural habitats. Once removed from the forest, rosewood logs and cut wood are currently impossible to identify to the species level and it is likewise impossible to determine their provenance. The DNA barcoding reference dataset presented here provides an important first step towards establishing a reliable molecular tool for the identification and tracking of traded *Dalbergia* species from Madagascar.

### Species discrimination

Our analysis revealed that the endemic Malagasy species of *Dalbergia* are not monophyletic but rather form at least two distinct monophyletic groups. These results are in agreement with those reported by Vatanparast *et al*. [50], supporting the inference of non-monophyly of Malagasy *Dalbergia*. The two Malagasy clades we recovered are divergent from African, American and Asian samples and indicate at least two independent colonization events of Madagascar.

Our results place the Malagasy samples of *Dalbergia* into four subgroups, three of which are well-supported (SG1, SG2 and SG4). With the exception of subgroup I, all subgroups encompass multiple species. The members of these subgroups currently cannot be distinguished at the species level using the barcoding markers considered here, but some of them can be distinguished morphologically. For example, SG2 comprises three species with distinct leaflet shapes and differences in leaflet length: *D*. *maritima* var. *pubescens* with small leaflets (0.5–1.6 cm), *D*. *louvelii* with intermediate leaflets (2–4 cm) and *D*. *madagascariensis* subsp. *antongilensis* with much larger leaflets (4–8 cm) [17]. Similarly, subgroup SG4 contains seven species that can be distinguished using a combination of morphology, ecology and geography. *Dalbergia baronii* and *D*. *monticola* both have small leaflets (0.5–1.7 cm) but the first occurs exclusively in low-altitude forests up to 150 m a.s.l. in eastern Madagascar whereas the latter is found in mid-altitude forests from 250–1600 m a.s.l. of eastern and central Madagascar. *Dalbergia pseudobaronii* has similar leaflets and spans the elevational ranges of *D*. *baronii* and *D*. *monticola*, from 50-1000m a.s.l., but has very distinct fruits [17]. In subgroup SG4, *D*. *normandii* has the largest leaflets (4–6 cm) and *D*. *orientalis* has leaflets of intermediate size (1.5–2.7 cm). These two species occur throughout the sampling area in northeastern Madagascar (sites 1–8, Fig 1). *Dalbergia mollis* has velvety pubescent leaflets with intermediate to large leaflet length (2–7 cm) and occurs in seasonally dry western forest up to 700m a.s.l. Finally, *D*. *greveana* also has intermediate to large sized leaflets (2.5–6 cm) and grows in seasonally dry western forests but is very distinct in its leaflet shape, making it easy to recognize even when voucher material is sterile.

Comparing our subgroups to those recovered by Vatanparast *et al*. [50] reveals several cases of concordance even though species sampling differed substantially between their study and ours. For the Malagasy species, their clade M1 corresponds to our monophyletic subgroup SG2 and their clade M3 closely matches our monophyletic subgroup SG4. Another shared finding is the separation of *Dalbergia bracteolata* from the other members of the genus in Madagascar, which is not surprising given that this species is thought to be originally from continental Africa [17]. Clade M2 of Vatanparast *et al*. [50] does not correspond to any of our subgroups, as it comprises species that were not included in our sampling. Our study revealed that *D*. *greveana* is more closely related to *D*. *baronii* than to *D*. *trichocarpa* as suggested in Vatanparast *et al*. [50]. The phylogenetic relationships inferred among non-Malagasy species in our study are similar to those reported by Vatanparast *et al*. [50] with one notable exception. Our analyses failed to resolve *D*. *spruceana* from Brazil as sister to all other species due to low branch support, which rendered its position unclear. The recent barcoding study of Hartvig *et al*. [33] focused on *Dalbergia* species from Asia and the largely non-overlapping species sampling precluded detailed comparisons between their results and ours. Together, these studies contribute to a better understanding of the evolutionary and phylogenetic relationships among *Dalbergia* species.

Species identification using DNA barcoding is known to be difficult among closely related species [51–53] and our identification success rates at the species level were indeed very low. In contrast, analyses above the species level provided good identification success. Thus, while reliable species identification is not possible with standard plastid DNA barcodes for Malagasy *Dalbergia*, groups of closely related species can be distinguished with high success and the Malagasy endemic *Dalbergia* species studied can readily be differentiated from non-Malagasy *Dalbergia* through the analysis of few diagnostic polymorphisms. The prospect of being able to differentiate successfully between Malagasy and non-Malagasy rosewoods and between groups of endemic species is an important result with regard to the enforcement of CITES regulations.

### Diagnostic polymorphisms

The three chloroplast markers analyzed in this study vary with respect to the number of sites that are informative for diagnostic purposes. To maximize barcoding efficiency in terms of both time and cost, we used the CAOS workbench to identify diagnostic sites that enable the delimitation of groups or subgroups of Malagasy *Dalbergia*. Our results revealed six diagnostic positions in the *trn*L (UAA) region for distinguishing between Malagasy and non-Malagasy *Dalbergia*. The advantage of the CAOS approach is that well-aligned insertions and deletions are also used as diagnostic sites. In our study, we found that the presence of *indel1* is diagnostic for the Malagasy group I and the absence of *indel2* is diagnostic for the Malagasy group II.

The *trn*L (UAA) region has repeatedly been found to have a high potential as a standard DNA barcoding marker [20,29,35,54,55]. It has not, however, overtaken other barcoding markers because it is difficult to sequence in some taxa [54], and while *trn*L (UAA) is known to provide resolution even among closely related species in some taxa [54,56,57], this marker exhibits low levels of divergence in others [58]. In the present study *trn*L (UAA) revealed more variation than *mat*K and *rbc*L but was not able to provide full species-level resolution among all Malagasy *Dalbergia* species investigated. The low resolution of chloroplast markers has been widely discussed [52,53,59,60] and is a key reason why finding a universal DNA barcode for land plants remains elusive [28,29].

### Potential applications

The aim of this study was to explore the utility of DNA barcoding for identification of Malagasy *Dalbergia* species and to initiate the development of a molecular reference dataset to help authorities and regulatory bodies with the identification of *Dalbergia* timber from Madagascar. DNA barcoding has been found useful for species identification in a wide diversity of research fields, ranging from pure ecology [27,61,62] to the verification of food declarations [63–65] and the identification of illegally traded species [23,66–69]. While many successful applications have been developed for animals, such as the African elephant [70–72], the lack of a universal barcode in plants has in many instances necessitated the development of case-specific identification systems. Several have already been developed to track important timber groups such as mahogany (*Swietenia*) [3], sapelli (*Entandrophragma*) [73], ramin (*Gonystylus*) [74] and oak (*Quercus*) [75]. More recent studies have included *Dalbergia* species mainly from Asia to test species identification and sample assignment [33–35]. These studies have demonstrated both the potential and limitations of DNA barcoding in timber tracking and have shown that it is particularly difficult to assign samples to exact geographic areas, despite the fact that this information is of utmost importance for law enforcement, as in situations where decisions need to be made based on whether logs originated from within a protected area.

Developing species identification systems for all CITES-listed species is of high priority, as discussed in Keong [76] and Höltken *et al*. [77]. In the case of *Dalbergia*, the ability to determine whether material originated from Madagascar or elsewhere is sufficient to identify illegally traded timber because all *Dalbergia* species of Malagasy origin are currently listed on CITES appendix II without any export quota. The DNA barcoding reference dataset developed here reveals that it is possible to determine whether rosewood of unclear or dubious origin came from Madagascar, which may be a valuable tool for the enforcement of CITES regulations. In order to realize the full potential of this tool, it will be necessary to expand the reference dataset to include all potentially harvestable species of *Dalbergia* from throughout Madagascar. Such a comprehensive reference dataset can then serve as the cornerstone for an international rosewood identification system that serves many authorities and plays an important role in efforts to combat the growing illegal exploitation and trade of Malagasy rosewoods. In addition to expanding the cpDNA reference dataset to include more species, it would also be worthwhile to explore whether nuclear DNA markers, such as microsatellites or single-nucleotide polymorphisms (SNPs), can be used to distinguish among *Dalbergia* taxa from Madagascar.

## Conclusions

DNA barcoding with standard chloroplast barcoding markers does not provide sufficient resolution to identify all *Dalbergia* species from Eastern Madagascar that were represented by multiple samples in this study. However, this method allows for efficient discrimination between Malagasy and non-Malagasy *Dalbergia* species, which may help enforce existing CITES regulations because all *Dalbergia* species from Madagascar are currently listed on CITES Appendix II.

## Supporting Information

S1 Table Sample information with BOLD accession numbers. List of taxon names and voucher information for 162 *Dalbergia* samples and the outgroup *Pterocarpus indicus*. Taxa are ordered alphabetically for all samples collected from Madagascar and below for non-Malagasy samples. The column “year” indicates when the samples were collected. Dashes indicate that a locality was not precisely known (e.g. DNA samples or living collections) in “sampling location”. Groups (I-IV) for non-Malagasy samples and subgroups (SG1-SG4) for Malagasy samples in Fig 2 are indicated in a separate column. Column “Voucher” gives the acronym of the public herbarium where vouchers are deposited or from the institutions that provided DNA samples. An asterisk (*) in column “Sample ID” indicates fertile vouchers.
(DOCX)

S2 Table Primer information for 15 tested chloroplast markers. (DOCX)
