# Supplementary material for: DNA Barcoding of Malagasy Rosewoods: Towards a Molecular Identification of CITES-Listed Dalbergia Species
Source: PLoS One. 2016 Jun 30;11(6):e0157881. doi: 10.1371/journal.pone.0157881 (PMC4928830; doi:10.1371/journal.pone.0157881)
Supplement: S1 Table — List of taxon names and voucher information for 162 Dalbergia samples and the outgroup Pterocarpus indicus. Taxa are ordered alphabetically for all samples collected from Madagascar and below for non-Malagasy samples. The column “year” indicates when the samples were collected. Dashes indicate that a locality was not precisely known (e.g. DNA samples or living collections) in “sampling location”. Groups (I-IV) for non-Malagasy samples and subgroups (SG1-SG4) for Malagasy samples in Fig 2 are indicated in a separate column. Column “Voucher” gives the acronym of the public herbarium where vouchers are deposited or from the institutions that provided DNA samples. An asterisk (*) in column “Sample ID” indicates fertile vouchers. (DOCX) [file pone.0157881.s001.docx]

**S1 Table**. **Sample information with BOLD accession numbers**. List of taxon names and voucher information for 162 *Dalbergia* samples and the outgroup *Pterocarpus indicus.* Taxa are ordered alphabetically for all samples collected from Madagascar and below for non-Malagasy samples. The column “year” indicates when the samples were collected. Dashes indicate that a locality was not precisely known (e.g. DNA samples or living collections) in “sampling location”. Groups (I-IV) for non-Malagasy samples and subgroups (SG1-SG4) for Malagasy samples in Fig 2 are indicated in a separate column. Column “Voucher” gives the acronym of the public herbarium where vouchers are deposited or from the institutions that provided DNA samples. An asterisk (*) in column “Sample ID” indicates fertile vouchers.

| **Taxon** | **Sample ID** | **Year** | **Voucher** | **Sampling location (Fig 1, Table 1)** | **Groups and subgroups (Fig 2)** | **BOLD accession numbers** |
| --- | --- | --- | --- | --- | --- | --- |
| Madagascar | | | | | | |
| *D. baronii* Baker | JA728 | 2010 | Z+ZT | 4 | SG4 | MADA024-14 |
| *D. baronii* Baker | JA740 | 2010 | Z+ZT | 4 | SG4 | MADA107-14 |
| *D. baronii* Baker | JA741 | 2010 | Z+ZT | 4 | SG4 | MADA108-14 |
| *D. baronii* Baker | K5 | 1988 | DNA bank | – | SG4 | MADA045-14 |
| *D. baronii* Baker | RBE2257 | 2014 | P | 12 | SG4 | MADA049-14 |
| *D. baronii* Baker | SH001 | 2011 | Z+ZT | 4 | SG4 | MADA126-14 |
| *D. baronii* Baker | SH006 | 2011 | Z+ZT | 4 | SG4 | MADA129-14 |
| *D. baronii* Baker | SH009 | 2011 | Z+ZT | 4 | SG4 | MADA058-14 |
| *D. baronii* Baker | SH010 | 2011 | Z+ZT | 4 | SG4 | MADA130-14 |
| *D. baronii* Baker | SH011 | 2011 | Z+ZT | 4 | SG4 | MADA131-14 |
| *D. baronii* Baker | SH017 | 2011 | Z+ZT | 4 | SG4 | MADA133-14 |
| *D. baronii* Baker | SH151 | 2011 | Z+ZT | 8 | SG4 | MADA068-14 |
| *D. baronii* Baker | SH225 | 2011 | Z+ZT | 6 | SG4 | MADA153-14 |
| *D. baronii* Baker | SH277 | 2011 | Z+ZT | 5 | SG4 | MADA159-14 |
| *D. baronii* Baker | SH640 | 2013 | Z+ZT | – | SG4 | MADA096-14 |
| *D. bracteolata* Baker | RIR2401 | 2014 | P | 14 | IV | MADA053-14 |
| *D. chapelieri* Baill. | EME1 | 2014 | P | 11 | SG1 | MADA017-14 |
| *D. chapelieri* Baill. | JA730 | 2010 | Z+ZT | 4 | SG1 | MADA025-14 |
| *D. chapelieri* Baill. | RBE2250 | 2014 | P | 12 | SG1 | MADA119-14 |
| *D. chapelieri* Baill. | RBE2266 | 2014 | P | 12 | SG1 | MADA050-14 |
| *D. chapelieri* Baill. | SH003 | 2011 | Z+ZT | 4 | SG1 | MADA128-14 |
| *D. chapelieri* Baill. | SH008 | 2011 | Z+ZT | 4 | SG1 | MADA057-14 |
| *D. chapelieri* Baill. | SH379 | 2011 | Z+ZT | 2 | SG1 | MADA174-14 |
| *D. chapelieri* Baill. | SH594 | 2013 | Z+ZT | 9 | SG1 | MADA089-14 |
| *D. chapelieri* Baill. | SH596 | 2013 | Z+ZT | 9 | SG1 | MADA090-14 |
| *D. chapelieri* Baill. | SH597 | 2013 | Z+ZT | 9 | SG1 | MADA198-14 |
| *D. chapelieri* Baill. | SH599 | 2013 | Z+ZT | 9 | SG1 | MADA199-14 |
| *D. chapelieri* Baill. | SH604* | 2013 | Z+ZT | 9 | SG1 | MADA091-14 |
| *D. greveana* Baill. | RIR2398 | 2014 | P | 14 | SG4 | MADA051-14 |
| *D. greveana* Baill. | RIR2468 | 2014 | P | 15 | SG4 | MADA123-14 |
| *D. greveana* Baill. | SH632* | 2013 | Z+ZT | – | SG4 | MADA094-14 |
| *D. greveana* Baill. | SH635 | 2013 | Z+ZT | – | SG4 | – |
| *D. louvelii* R. Vig. | RBE2247 | 2014 | P | 12 | SG2 | MADA117-14 |
| *D. madagascariensis* Vatke subsp. *antongilensis* Bosser & R. Rabev. | SH030 | 2011 | Z+ZT | 7 | SG2 | MADA061-14 |
| *D. madagascariensis* Vatke subsp. *antongilensis* Bosser & R. Rabev. | SH038 | 2011 | Z+ZT | 7 | SG2 | MADA136-14 |
| *D. madagascariensis* Vatke subsp. *antongilensis* Bosser & R. Rabev. | SH041 | 2011 | Z+ZT | 7 | SG2 | MADA137-14 |
| *D. madagascariensis* Vatke subsp. *antongilensis* Bosser & R. Rabev. | SH053 | 2011 | Z+ZT | 7 | SG2 | MADA139-14 |
| *D. madagascariensis* Vatke subsp. *antongilensis* Bosser & R. Rabev. | SH076 | 2011 | Z+ZT | 7 | SG2 | MADA066-14 |
| *D. madagascariensis* Vatke subsp. *antongilensis* Bosser & R. Rabev. | SH101 | 2011 | Z+ZT | 7 | SG2 | MADA142-14 |
| *D. madagascariensis* Vatke subsp. *antongilensis* Bosser & R. Rabev. | SH108 | 2011 | Z+ZT | 8 | SG2 | MADA067-14 |
| *D. madagascariensis* Vatke subsp. *antongilensis* Bosser & R. Rabev. | SH146 | 2011 | Z+ZT | 8 | SG2 | MADA143-14 |
| *D. madagascariensis* Vatke subsp. *antongilensis* Bosser & R. Rabev. | SH152 | 2011 | Z+ZT | 8 | SG2 | MADA145-14 |
| *D. madagascariensis* Vatke subsp. *antongilensis* Bosser & R. Rabev. | SH155 | 2011 | Z+ZT | 8 | SG2 | MADA147-14 |
| *D. madagascariensis* Vatke subsp. *antongilensis* Bosser & R. Rabev. | SH156 | 2011 | Z+ZT | 8 | SG2 | MADA069-14 |
| *D. madagascariensis* Vatke subsp. *antongilensis* Bosser & R. Rabev. | SH189 | 2011 | Z+ZT | 6 | SG2 | MADA070-14 |
| *D. maritima* R. Vig. var*. pubescens* Bosser & R. Rabev. | JA717 | 2010 | Z+ZT | 4 | SG2 | MADA023-14 |
| *D. maritima* R. Vig. var*. pubescens* Bosser & R. Rabev. | JA720 | 2010 | Z+ZT | 4 | SG2 | MADA104-14 |
| *D. maritima* R. Vig. var*. pubescens* Bosser & R. Rabev. | JA743 | 2010 | Z+ZT | 4 | SG2 | MADA110-14 |
| *D. maritima* R. Vig. var*. pubescens* Bosser & R. Rabev. | SH047 | 2011 | Z+ZT | 7 | SG2 | MADA062-14 |
| *D. maritima* R. Vig. var*. pubescens* Bosser & R. Rabev. | SH050 | 2011 | Z+ZT | 7 | SG2 | MADA063-14 |
| *D. maritima* R. Vig. var*. pubescens* Bosser & R. Rabev. | SH071 | 2011 | Z+ZT | 7 | SG2 | MADA065-14 |
| *D. maritima* R. Vig. var*. pubescens* Bosser & R. Rabev. | SH200 | 2011 | Z+ZT | 6 | SG2 | MADA071-14 |
| *D. maritima* R. Vig. var*. pubescens* Bosser & R. Rabev. | SH232 | 2011 | Z+ZT | 6 | SG2 | MADA155-14 |
| *D. maritima* R. Vig. var*. pubescens* Bosser & R. Rabev. | SH276 | 2011 | Z+ZT | 5 | SG2 | MADA158-14 |
| *D. mollis* Bosser & R.Rabev. | RIR2471 | 2014 | P | 15 | SG4 | MADA056-14 |
| *D. monticola* Bosser & R. Rabev. | RAF12 | 2014 | P | 13 | SG3 | MADA113-14 |
| *D. monticola* Bosser & R. Rabev. | K23 | 1992 | DNA bank | – | SG4 | MADA033-14 |
| *D. monticola* Bosser & R. Rabev. | SH285 | 2011 | Z+ZT | 3 | SG4 | MADA162-14 |
| *D. monticola* Bosser & R. Rabev. | SH290 | 2011 | Z+ZT | 3 | SG4 | MADA075-14 |
| *D. monticola* Bosser & R. Rabev. | SH293 | 2011 | Z+ZT | 3 | SG4 | MADA076-14 |
| *D. monticola* Bosser & R. Rabev. | SH349 | 2011 | Z+ZT | 3 | SG4 | MADA169-14 |
| *D. monticola* Bosser & R. Rabev. | SH394 | 2011 | Z+ZT | 2 | SG4 | MADA079-14 |
| *D. monticola* Bosser & R. Rabev. | SH397 | 2011 | Z+ZT | 2 | SG4 | MADA176-14 |
| *D. monticola* Bosser & R. Rabev. | SH407 | 2011 | Z+ZT | 2 | SG4 | MADA177-14 |
| *D. monticola* Bosser & R. Rabev. | SH482 | 2011 | Z+ZT | 1 | SG4 | MADA183-14 |
| *D. monticola* Bosser & R. Rabev. | SH486 | 2011 | Z+ZT | 1 | SG4 | MADA082-14 |
| *D. monticola* Bosser & R. Rabev. | SH556* | 2013 | Z+ZT | – | SG3 | MADA184-14 |
| *D. monticola* Bosser & R. Rabev. | SH560 | 2013 | Z+ZT | 9 | SG3 | MADA185-14 |
| *D. monticola* Bosser & R. Rabev. | SH561 | 2013 | Z+ZT | 9 | SG3 | MADA083-14 |
| *D. monticola* Bosser & R. Rabev. | SH563 | 2013 | Z+ZT | 9 | SG3 | MADA187-14 |
| *D. monticola* Bosser & R. Rabev. | SH564 | 2013 | Z+ZT | 9 | SG3 | MADA084-14 |
| *D. monticola* Bosser & R. Rabev. | SH565* | 2013 | Z+ZT | 9 | SG3 | MADA188-14 |
| *D. monticola* Bosser & R. Rabev. | SH566 | 2013 | Z+ZT | 9 | SG3 | MADA085-14 |
| *D. monticola* Bosser & R. Rabev. | SH568 | 2013 | Z+ZT | 9 | SG3 | MADA189-14 |
| *D. monticola* Bosser & R. Rabev. | SH570 | 2013 | Z+ZT | 9 | SG3 | MADA190-14 |
| *D. monticola* Bosser & R. Rabev. | SH571 | 2013 | Z+ZT | 9 | SG3 | MADA191-14 |
| *D. monticola* Bosser & R. Rabev. | SH573 | 2013 | Z+ZT | 9 | SG3 | MADA086-14 |
| *D. monticola* Bosser & R. Rabev. | SH574 | 2013 | Z+ZT | 9 | SG3 | MADA192-14 |
| *D. monticola* Bosser & R. Rabev. | SH582 | 2013 | Z+ZT | 9 | SG3 | MADA193-14 |
| *D. monticola* Bosser & R. Rabev. | SH583 | 2013 | Z+ZT | 9 | SG3 | MADA194-14 |
| *D. monticola* Bosser & R. Rabev. | SH585 | 2013 | Z+ZT | 9 | SG3 | MADA195-14 |
| *D. monticola* Bosser & R. Rabev. | SH587 | 2013 | Z+ZT | 9 | SG3 | MADA196-14 |
| *D. monticola* Bosser & R. Rabev. | SH588 | 2013 | Z+ZT | 9 | SG3 | MADA087-14 |
| *D. monticola* Bosser & R. Rabev. | SH589 | 2013 | Z+ZT | 9 | SG3 | MADA197-14 |
| *D. monticola* Bosser & R. Rabev. | SH591 | 2013 | Z+ZT | 9 | SG3 | MADA088-14 |
| *D. monticola* Bosser & R. Rabev. | SH603 | 2013 | Z+ZT | 9 | SG3 | MADA200-14 |
| *D. monticola* Bosser & R. Rabev. | SH609 | 2013 | Z+ZT | 10 | SG3 | MADA201-14 |
| *D. monticola* Bosser & R. Rabev. | SH610 | 2013 | Z+ZT | 10 | SG3 | MADA202-14 |
| *D. monticola* Bosser & R. Rabev. | SH611 | 2013 | Z+ZT | 10 | SG3 | MADA203-14 |
| *D. monticola* Bosser & R. Rabev. | SH614 | 2013 | Z+ZT | 10 | SG3 | MADA204-14 |
| *D. monticola* Bosser & R. Rabev. | SH615 | 2013 | Z+ZT | 10 | SG3 | MADA205-14 |
| *D. monticola* Bosser & R. Rabev. | SH617 | 2013 | Z+ZT | 10 | SG3 | MADA206-14 |
| *D. monticola* Bosser & R. Rabev. | SH618 | 2013 | Z+ZT | 10 | SG3 | MADA207-14 |
| *D. monticola* Bosser & R. Rabev. | SH620 | 2013 | Z+ZT | 10 | SG3 | MADA208-14 |
| *D. monticola* Bosser & R. Rabev. | SH621 | 2013 | Z+ZT | 10 | SG3 | MADA209-14 |
| *D. monticola* Bosser & R. Rabev. | SH622 | 2013 | Z+ZT | 10 | SG3 | MADA210-14 |
| *D. monticola* Bosser & R. Rabev. | SH623 | 2013 | Z+ZT | 10 | SG3 | MADA211-14 |
| *D. monticola* Bosser & R. Rabev. | SH624 | 2013 | Z+ZT | 10 | SG3 | MADA092-14 |
| *D. monticola* Bosser & R. Rabev. | SH626 | 2013 | Z+ZT | 10 | SG3 | MADA093-14 |
| *D. monticola* Bosser & R. Rabev. | SH638 | 2013 | Z+ZT | – | SG4 | MADA095-14 |
| *D. normandii* Bosser & R. Rabev. | JA739 | 2010 | Z+ZT | 4 | SG4 | MADA106-14 |
| *D. normandii* Bosser & R. Rabev. | JL026 | 2010 | ETHZ | – | SG4 | MADA112-14 |
| *D. normandii* Bosser & R. Rabev. | SH012 | 2011 | Z+ZT | 4 | SG4 | MADA059-14 |
| *D. normandii* Bosser & R. Rabev. | SH233 | 2011 | Z+ZT | 6 | SG4 | MADA073-14 |
| *D. normandii* Bosser & R. Rabev. | SH238 | 2011 | Z+ZT | 6 | SG4 | MADA156-14 |
| *D. normandii* Bosser & R. Rabev. | SH334 | 2011 | Z+ZT | 3 | SG4 | MADA077-14 |
| *D. occulta* Bosser & R. Rabev. | SH452 | 2011 | Z+ZT | 1 | SG2 | MADA080-14 |
| *D. orientalis* Bosser & R. Rabev. | SH015 | 2011 | Z+ZT | 4 | SG4 | MADA060-14 |
| *D. orientalis* Bosser & R. Rabev. | SH051 | 2011 | Z+ZT | 7 | SG4 | MADA064-14 |
| *D. orientalis* Bosser & R. Rabev. | SH218 | 2011 | Z+ZT | 6 | SG4 | MADA072-14 |
| *D. orientalis* Bosser & R. Rabev. | SH226 | 2011 | Z+ZT | 6 | SG4 | MADA154-14 |
| *D. orientalis* Bosser & R. Rabev. | SH239 | 2011 | Z+ZT | 6 | SG4 | MADA074-14 |
| *D. orientalis* Bosser & R. Rabev. | SH265 | 2011 | Z+ZT | 5 | SG4 | MADA157-14 |
| *D. orientalis* Bosser & R. Rabev. | SH280 | 2011 | Z+ZT | 5 | SG4 | MADA161-14 |
| *D. orientalis* Bosser & R. Rabev. | SH335 | 2011 | Z+ZT | 3 | SG4 | MADA078-14 |
| *D. pseudobaronii* R. Vig. | SH387 | 2011 | Z+ZT | 2 | SG4 | MADA175-14 |
| *D. pseudobaronii* R. Vig. | SH479 | 2011 | Z+ZT | 1 | SG4 | MADA081-14 |
| *D. pseudobaronii* R. Vig. | SH481 | 2011 | Z+ZT | 1 | SG4 | MADA182-14 |
| *D. purpurascens* Baill. | RIR2410 | 2014 | P | 14 | SG3 | MADA054-14 |
| *D. trichocarpa* Baker | RIR2470 | 2014 | P | 15 | SG3 | MADA055-14 |
| *D. urschii* Bosser & R. Rabev. | RIR2458 | 2014 | P | 14 | SG3 | MADA122-14 |
| Outside Madagascar | | | | | | |
| *D. arbutifolia* Baker | AF15 | NA | BNRH | Sofala, Mozambique | IV | MADA006-14 |
| *D. arbutifolia* Baker | AF19 | 2010 | BNRH | Sofala, Mozambique | IV | MADA008-14 |
| *D. arbutifolia* Baker | FTG2 | 2014 | FTG | Fairchild Tropical Botanical Garden, Florida, USA | IV | MADA019-14 |
| *D. armata* E. Mey. | AF10 | NA | BNRH | Mpumalanga, South Africa | IV | MADA002-14 |
| *D. armata* E. Mey. | AF11 | 2010 | BNRH | Mpumalanga, South Africa | IV | MADA003-14 |
| *D. armata* E. Mey. | AF8 | 2007 | BNRH | Mpumalanga, South Africa | IV | MADA015-14 |
| *D. armata* E. Mey. | K4 | NA | DNA bank | South Africa | IV | MADA043-14 |
| *D. boehmii* Taub. | AF14 | NA | BNRH | Sofala, Mozambique | III | MADA005-14 |
| *D. boehmii* Taub. | AF17 | NA | BNRH | Sofala, Mozambique | III | MADA007-14 |
| *D. cearensis* Ducke | K8 | 1985 | DNA bank | Brazil | IV | MADA046-14 |
| *D. cearensis* Ducke | K9 | 1995 | DNA bank | Brazil | IV | MADA047-14 |
| *D. cochinchinensis* Pierre | K10 | 1999 | DNA bank | Chachoengsao, Thailand | III | MADA026-14 |
| *D. dongnaiensis* Pierre | K12 | 1946 | DNA bank | Tripagodas, Thailand Kwae Noi River Basin Exp., Birmese border | III | MADA027-14 |
| *D. ecastophyllum* (L.) Taub. | FTG1 | 2014 | FTG | Fairchild Tropical Botanical Garden, Florida, USA | IV | MADA018-14 |
| *D. granadillo* Pittier | K15 | 1981 | DNA bank | Mexico | III | MADA028-14 |
| *D. hainanensis* Merr. & Chun | K17 | 2005 | DNA bank | Hainan, China | IV | MADA029-14 |
| *D. hupeana* Hance | K18 | 2005 | DNA bank | Mt. Guting, Luzhai Co., Guangxi province, China | III | MADA030-14 |
| *D. melanoxylon* Guill. & Perr. | AF13 | 2010 | BNRH | Sofala, Mozambique | III | MADA004-14 |
| *D. melanoxylon* Guill. & Perr. | AF4 | 2005 | BNRH | Mpumalanga, South Africa | III | MADA011-14 |
| *D. melanoxylon* Guill. & Perr. | AF5 | NA | BNRH | Mpumalanga, South Africa | III | MADA012-14 |
| *D. melanoxylon* Guill. & Perr. | AF6 | NA | BNRH | Mpumalanga, South Africa | III | MADA013-14 |
| *D. melanoxylon* Guill. & Perr. | AF7 | NA | BNRH | Mpumalanga, South Africa | III | MADA014-14 |
| *D. melanoxylon* Guill. & Perr. | B2 | 2012 | Botanical Garden Meise, Belgium | Africa | III | MADA016-14 |
| *D. melanoxylon* Guill. & Perr. | K22 | 1964 | DNA bank | Tanzania | III | MADA032-14 |
| *D. multijuga* E. Mey. | AF1 | 2010 | BNRH | KwaZulu-Natal, South Africa | IV | MADA001-14 |
| *D. nigrescens* Kurz | K25 | 1968 | DNA bank | Eastern Part of Khao Yai NP, Pak Thong Chai, Thailand | III | MADA034-14 |
| *D. obovata* E. Mey. | AF2 | 2010 | BNRH | KwaZulu-Natal, South Africa | IV | MADA009-14 |
| *D. obovata* E. Mey. | AF3 | NA | BNRH | Kwazulu-Natal, South Africa | IV | MADA010-14 |
| *D. odorifera* T. C. Chen | K26 | 2005 | DNA bank | Hainan, China | IV | MADA035-14 |
| *D. oliveri* Prain | HTT4 | 2013 | ETHZ | Vietnam | III | MADA020-14 |
| *D. oliveri* Prain | HTT5 | 2013 | ETHZ | Vietnam | III | MADA021-14 |
| *D. oliveri* Prain | HTT6 | 2013 | ETHZ | Vietnam | III | MADA022-14 |
| *D. ovata* Benth. | K28 | 1949 | DNA bank | Rangoon, Mayangone, Burma | III | MADA036-14 |
| *D. sericea* G. Don | K33 | 1979 | DNA bank | Mangde Chu Valley N of Shamgong, Bhutan | III | MADA038-14 |
| *D. sissoo* Roxb. ex DC. | K2 | NA | DNA bank | NW Himalayas, India | IV | MADA031-14 |
| *D. sissoo* Roxb. ex DC. | K35 | 1985 | DNA bank | India | IV | MADA039-14 |
| *D. spruceana* Benth. | K3 | NA | DNA bank | Para, Brazil | IV | MADA037-14 |
| *D. spruceana* Benth. | K36 | 1996 | DNA bank | Bolivia | IV | MADA040-14 |
| *D. stipulacea* Roxb. | K38 | 1952 | DNA bank | Bhutan | III | MADA041-14 |
| *D. tucurensis* Donn. Sm. | K39 | 1991 | DNA bank | Guatemala | IV | MADA042-14 |
| *D. yunnanensis* Franch. | K40 | 1981 | DNA bank | Sino-British Exp. to Cangshan, China | IV | MADA044-14 |
| *Pterocarpus indicus* Willd. | 118737383 | 2011 | GenBank | Puerto Rico |  | matK: JN083546.1  rbcL: JN083725.1  trnL: AF208953.1 |

*Acronyms (Voucher):* BNRH=Buffelskloof Herbarium, Mpumalanga, South Africa; ETHZ=ETH Zurich, Zurich, Switzerland; FTG=Fairchild Tropical Botanical Garden, Florida, USA; P=Muséum National d'Histoire Naturelle, Herbier National, Paris, France; Z+ZT=Zürcher Herbarien, Zurich, Switzerland
