# Supplementary material for: DNA Barcoding of Malagasy Rosewoods: Towards a Molecular Identification of CITES-Listed Dalbergia Species
Source: PLoS One. 2016 Jun 30;11(6):e0157881. doi: 10.1371/journal.pone.0157881 (PMC4928830; doi:10.1371/journal.pone.0157881)
Supplement: S2 Table — (DOCX) [file pone.0157881.s002.docx]

| **Region** | **Primer_fwd** | **Primer_rev** | **Primer sequence (5' - 3')** | **Source** |
| --- | --- | --- | --- | --- |
| accD | accD1_f | accD3_r | AGTATGGGATCCGTAGTAGG/ TTTAAAGGATTACGTGGTAC | [1] |
| atpF | atpF f | atpH r | ACTCGCACACACTCCCTTTCC/ GCTTTTATGGAAGCTTTAACAAT | [2] |
| ITS | ITS_1f | ITS_4r | TCCGTAGGTGAACCTGCGG/ TCCTCCGCTTATTGATATGC | [3] |
| matK | matK_3F_KIM f | matK_1R_KIM r | CGTACAGTACTTTTGTGTTTACGAG/ ACCCAGTCCATCTGGAAATCTTGGTTC | [2] |
| matK | matK4 | matK19 | CCTTCGATACTGGGTGAAAGAT/ CCAGACCGGCTTACTAATGGG | [4] |
| ndhJ | ndhJ1_f | ndhJ4_r | CATAGATCTTTGGGCTTYGA /TCAATGAGCATCTTGTATTTC | [1] |
| psbA-trnH | psbA_1f_SH | trnH_2r_SH | GTTATGCATGAACGTAATGCTC/ ACGGGAATTGAACCCGCGCA | [2] |
| psbA-trnH | psbA_1f | trnH_1r | GTTATGCATGAACGTAATGCTC/ CGCGCATGGTGGATTCACAATCC | [5] |
| rbcL | rbcL_f_SH | rbcLa_R | GTAAAATCAAGTCCACCRCG/ ATGTCACCACAAACAGAGACTAAAGC | [2] |
| rbcL | rbcL1F | rbcL724R | ATGTCACCACAAACAGAAAC/ TCGCATGTACCTGCAGTAGC | [6] |
| rpoB2 | rpoB2_f | rpoB2_r | ATGCAACGTCAAGCAGTTCC/ CCGTATGTGAAAAGAAGTATA | [1] |
| rpoC1 | rpoC1_2_f | rpoC1_4_r | GGCAAAGAGGGAAGATTTCG/ CCATAAGCATATCTTGAGTTGG | [1] |
| trnL (UAA) | trnL_cf | trnL_dr | CGAAATCGGTAGACGCTACG/  GGGGATAGAGGGACTTGAAC | [7] |
| trnL-trnF | trnL_ef | trnF_f | GGTTCAAGTCCCTCTATCCC/ ATTTGAACTGGTGACACGAG | [7] |
| YCF | YCF5_2_f | YCF5_4_r | ACTTTAGAGCATATATTAACTC/ CCCAATACCATCATACTTAC | [1] |

**S2 Table**. Primer information for 15 tested chloroplast markers.

References

1. Chase MW, Cowan RS, Hollingsworth PM, van den Berg C, Madrinan S, Petersen G, et al. A proposal for a standardised protocol to barcode all land plants. Taxon. 2007;56: 295–299.

2. CBOL Plant Working Group. A DNA barcode for land plants. P Natl Acad Sci Usa. 2009;106: 12794–12797. doi:10.1073/pnas.0905845106

3. White T, Bruns T, Lee S, Taylor J. Amplification and direct sequencing of fungal ribosomal RNA genes for phylogenetics. In: White T, Innis M, Sninsky JJ, editors. PCR protocols: A guide to methods and applications. Academic Press; 1990. pp. 315–322.

4. Wojciechowski MF, Lavin M, Sanderson MJ. A phylogeny of legumes (Leguminosae) based on analysis of the plastid *mat*K gene resolves many well-supported subclades within the family. Am J Bot. 2004;91: 1846–1862. doi:10.3732/ajb.91.11.1846

5. Sang T, Crawford DJ, Stuessy TF. Chloroplast DNA phylogeny, reticulate evolution, and biogeography of *Paeonia* (Paeoniaceae). Am J Bot. 1997;84: 1120–1136.

6. Fay MF, Bayer C, Alverson WS, de Bruijn AY, Chase MW. Plastid *rbc*L sequence data indicate a close affinity between *Diegodendron* and *Bixa*. Taxon. 1998;47: 43–50. Available: http://gateway.webofknowledge.com/gateway/Gateway.cgi?GWVersion=2&SrcAuth=mekentosj&SrcApp=Papers&DestLinkType=FullRecord&DestApp=WOS&KeyUT=000072326500003

7. Taberlet P, Gielly L, Pautou G, Bouvet J. Universal primers for amplification of three non-coding regions of chloroplast DNA. Plant Mol Biol. 1991;17: 1105–1109. Available: http://link.springer.com/article/10.1007%2FBF00037152
